# Establishing a hybrid system for cardiac patients -developments and safety issues

**DOI:** 10.1186/1532-429X-15-S1-E22

**Published:** 2013-01-30

**Authors:** Melanie Jones, Juliet Semple, Tim Dent, Alex Pitcher, Malenka M Bissell, Elizabeth M Tunnicliffe, Hayley Harvey, Paul Leeson, Rajesh Kharbanda, Erica Dall'Armellina, Robin P Choudhury

**Affiliations:** 1 Acute Vascular Imaging Centre, Oxford University, Oxford, UK; 2 Cardiovascular Medicine, Oxford University, Oxford, UK; 3 Cardiac Medicine, Oxford University Hospitals NHS Trust, Oxford, UK

## Background

CMR is the preeminent imaging technique to assess LV function and myocardial injury. With the advent of combined angiography / CMR suites, there is an opportunity to perform combined invasive hemodynamic evaluation and treatments further informed by CMR imaging. However, key considerations are safety issues in potentially unstable patients in the context of MR and X-ray hazards. Here, we report our first experience on the feasibility of combined cardiac studies in this setting.

## Methods

**Figure 1:**
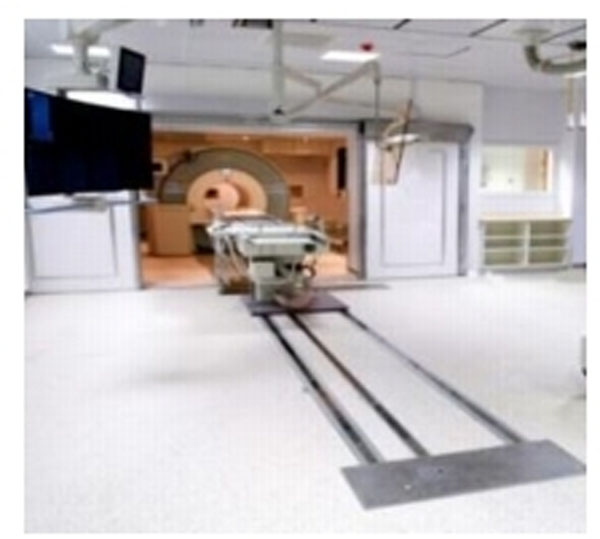


**Figure 2:**
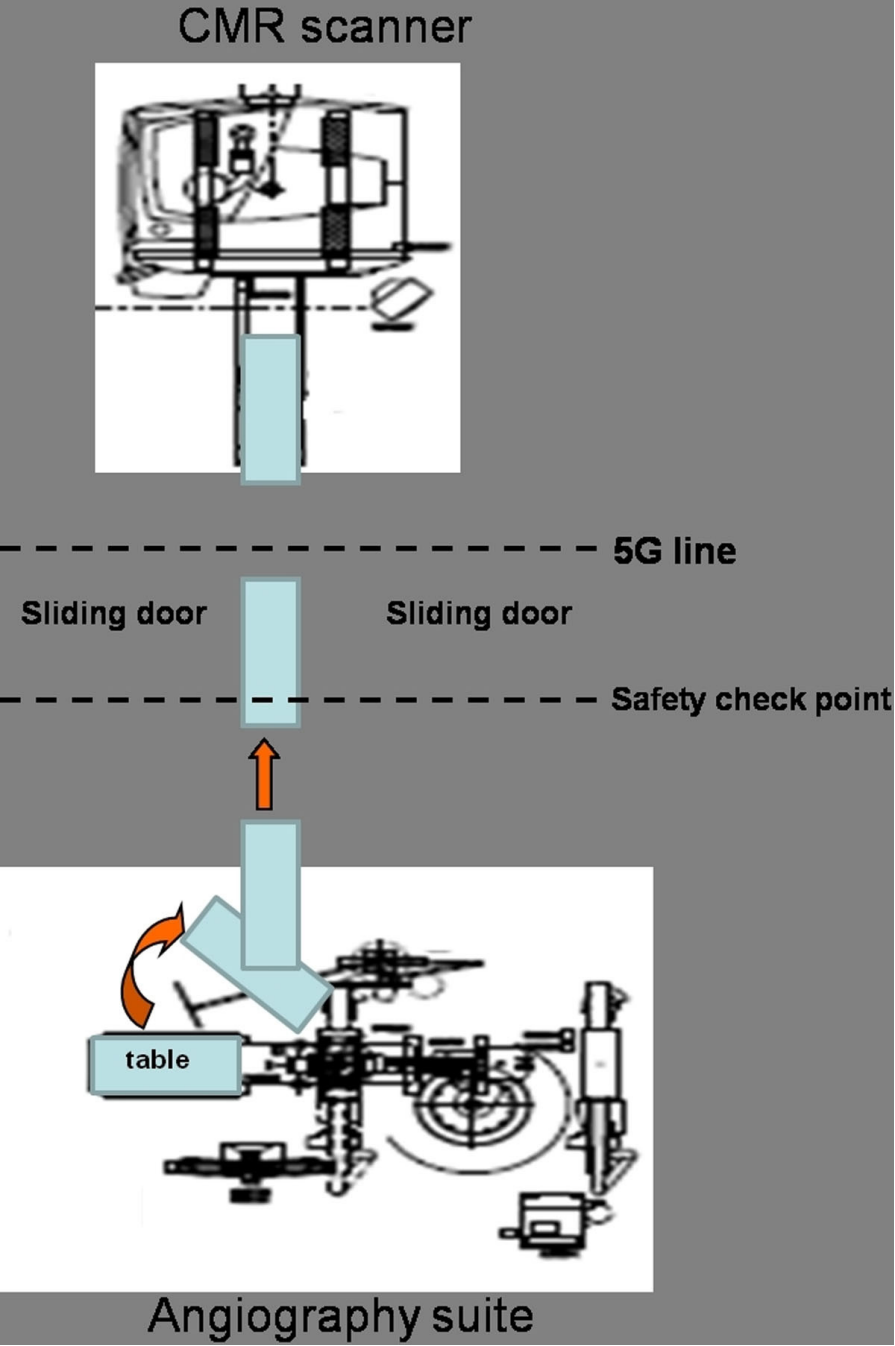


Twelve patients underwent the combined invasive angiography and CMR (Siemens Biplane Artis Zee angiography suite conjoined to a 3T Siemens Verio MR scanner). This system is equipped with a custom built direct transfer on the Angio-MR MIYABI table system, to transfer the patients from the angiographic suite into the scanner with seamless movement, minimal disruption and invasive intra-arterial monitoring via femoral / radial artery lines in situ (see Figures 1 and 2). The study protocol included invasive blood pressure measurements in the aorta in the angiographic suite using magnet-safe non-braided catheters (AngioDynamics, Cambridge, UK.) via a femoral approach and subsequently transfer of the patient into the scanner for CMR imaging. Safety checklists in transferring the patient from the cath lab to the CMR environment were in place. We employed a hierarchical system of safety measures that are applicable for units of this sort: from building design; safety feature installation to procedural design and training of non-specialist nursing and support staff.

## Results

Out of 12 patients recruited, 4 had contraindications to CMR; the remaining 8 (6M: 2F, aged between 48-79 yrs) successfully underwent the complete angiography and CMR imaging using the MIYABI function. No adverse events occurred. The mean total procedure time was 105 ± 30 minutes - comprising 60 ± 20 minutes in the cath lab and 45 ± 10 minutes in CMR. The core staff comprised: (1) Cath. Lab: 1 interventionalist, 1 physiologist, 1 X-ray radiographer, 2 nurses in the angio suite; and (2) CMR: 1 CMR radiographer to oversee MRI safety and 2 members of staff during the MIYABI transfer; 1 scan operator and 2 other members of staff in the MR control room.

## Conclusions

We report a structural and procedural design for hybrid CMR and invasive arteriography at 3T.

## Funding

BRC Heart theme

